# Tubarial glands as hidden culprits in post-viral Eustachian tube dysfunction mimicking otosclerosis: a call for advanced imaging integration

**DOI:** 10.1097/MS9.0000000000004594

**Published:** 2025-12-15

**Authors:** Abdullah Imtiaz, Muhammad Talha, Mahnoor Fatima, Omar Abdullah Gill, Muneeb Saifullah

**Affiliations:** a Department of Medicine, Wah Medical College, Wah Cantt, Pakistan; b Department of Medicine, King Edward Medical University, Lahore, Pakistan; c Department of Medicine, Shaheed Ziaur Rahman Medical College, Rajshahi Medical University, Bogura, Bangladesh

*To the Editor,*

Tubarial glands are a recently discovered type of salivary gland that lies adjacent to the torus tubarius and the nasopharynx on its lateral aspect near the Eustachian tube. The tubarial glands are thought to lubricate the oropharyngeal and nasopharyngeal mucosae, and if the lubrication is disturbed, possibly with viral infections, these glands can be a contributing factor in Eustachian tube dysfunction, which is characterized by impaired ventilation and pressure regulation in the middle ear and is associated with symptoms including conductive hearing loss. The close anatomical proximity of the tubarial glands to the Eustachian tube strongly suggests that their role in Eustachian Tube Dysfunction (ETD) pathophysiology warrants systematic investigation in clinical practice^[1,2]^. This is in line with the TITAN Guidelines on the need for transparency in AI use in healthcare^[3]^.

The presence of Eustachian tube dysfunction symptoms mimicking otosclerosis in patients recovering from a respiratory viral infection raises questions about the diagnosis. Otosclerosis is characterized by abnormal bone remodeling of the otic capsule, resulting in conductive hearing loss. Viral infection involving ETD as a result of tubarial gland inflammatory process or dysfunction can lead to a quite similar occurring clinical scenario (conductive hearing loss, tympanic membrane moving normally during pneumatic otoscopy, and Rinne test negative), thus causing problems in diagnosis and possibly resulting in delayed or ineffective treatment^[4,5]^.

Although tubarial glands may be involved in the presentation of ETD, failure to recognize them as such may produce suboptimal treatment outcomes of auditory symptoms: recurrent or persistent conductive hearing loss that might not respond to therapies designed to address middle ear pathologies or ossicular chain abnormalities. Specifically addressing tubarial gland inflammation or secretory dysfunction is therefore crucial; otherwise, neglecting this hidden culprit might lead to the recurrence of symptoms and a decrease in the patient’s quality of life. The consideration of tubarial gland involvement in modern auditory therapeutic protocols could lead to less need for multiple interventions and treatment resistance in the management of post-viral conductive hearing loss^[6,7]^.

Advanced imaging techniques offer interesting possibilities for enhancing detection capabilities and the comprehension of tubarial gland involvement during ETD. Conventional imaging techniques such as CT and standard MRI are not very successful in visualizing the glands. In comparison, PET/CT imaging using prostate-specific membrane antigen (PSMA) ligands provides remarkable visualization of the tubarial glands because of their extremely high physiological PSMA expression. The new MRI sequences, along with PSMA-targeted contrast agents, might allow a non-invasive, high-resolution evaluation of glandular inflammation or dysfunction, which would pave the way for personalized anti-inflammatory treatment and more accurate differentiation between post-viral ETD and true otosclerosis^[2]^.

To sum up, tubarial glands are a main cause of post-viral, non-infectious Eustachian tube dysfunction that can very well imitate otosclerosis and thus lead to a situation where it’s hard to tell the right cure or diagnosis. It is especially through the PSMA ligand-enhanced PET/CT and MRI imaging that integration of advanced imaging modalities in the diagnostic workflow may detect the glandular disease at an early stage and point out the right treatment. Due to the considerable effect of this diagnostic error on the clinic, it is a necessity for more studies to be done so as to reveal the pathophysiological mechanisms of tubarial gland dysfunction after viral infection and to come up with the strategies based on evidence that are capable of maximizing the benefits for the post-viral conductive hearing loss patients.

## Data Availability

Not applicable to this study.
